# Disseminated Musculoskeletal Coccidioidomycosis in an Immunocompetent Host: A Rare Case Requiring Extensive Spinal and Pelvic Surgery

**DOI:** 10.7759/cureus.87716

**Published:** 2025-07-11

**Authors:** Elangovan Raman, Sorabh Sharma

**Affiliations:** 1 Department of Internal Medicine, Madras Medical College and Rajiv Gandhi Government General Hospital, Chennai, IND; 2 Department of Internal Medicine, University of Arizona College of Medicine - Tucson, Tucson, USA

**Keywords:** african american male, cervical discitis, disseminated coccidioides, immunocompetent adult, spinal coccidioidomycosis

## Abstract

Coccidioidomycosis, also known as “Valley Fever,” is an endemic fungal infection caused by *Coccidioides* species, typically presenting as a respiratory illness. Disseminated disease, particularly with extensive skeletal involvement, is rare and typically seen in immunocompromised individuals. We report a unique and severe case of disseminated musculoskeletal coccidioidomycosis in a previously healthy immunocompetent 33-year-old African American man, involving multiple spinal levels, pelvic bones, and the right chest wall. The patient required multiple orthopedic surgeries, including anterior and posterior spinal decompression and fusion, bilateral sacroiliac joint debridement, and pelvic fixation. The diagnosis was confirmed via bone biopsy demonstrating Coccidioides spherules. Despite antifungal therapy, his disease progressed, necessitating escalation of treatment from fluconazole to isavuconazole and liposomal amphotericin B, followed by initiation of fosmanogepix as part of the approved treatment regimen. This case highlights the importance of high clinical suspicion, aggressive surgical intervention, and multidisciplinary management in complex disseminated coccidioidomycosis, even in immunocompetent hosts.

## Introduction

Coccidioidomycosis is a dimorphic fungal infection endemic to the southwestern United States, parts of Mexico, and Central and South America [1,2]. Although most infections remain localized to the lungs and resolve spontaneously, approximately 1% of cases disseminate to extrapulmonary sites, including the skin, central nervous system, and bones [3]. Disseminated musculoskeletal coccidioidomycosis is an uncommon manifestation and is usually associated with immunosuppression, diabetes, pregnancy, or ethnic susceptibility, including individuals of African descent [4]. Skeletal involvement commonly affects the vertebrae and pelvis, often mimicking malignancy, tuberculosis, or pyogenic infections [5]. Diagnosis requires high clinical suspicion and is confirmed via biopsy, culture, or serologic testing [6-8]. Management includes long-term antifungal therapy and often surgical intervention [9]. We present a complex and aggressive case of disseminated coccidioidomycosis with vertebral and pelvic osteomyelitis, epidural phlegmon, and a chest wall mass in an immunocompetent African American male patient, requiring multiple surgeries and prolonged antifungal therapy.

## Case presentation

**Figure 1:**
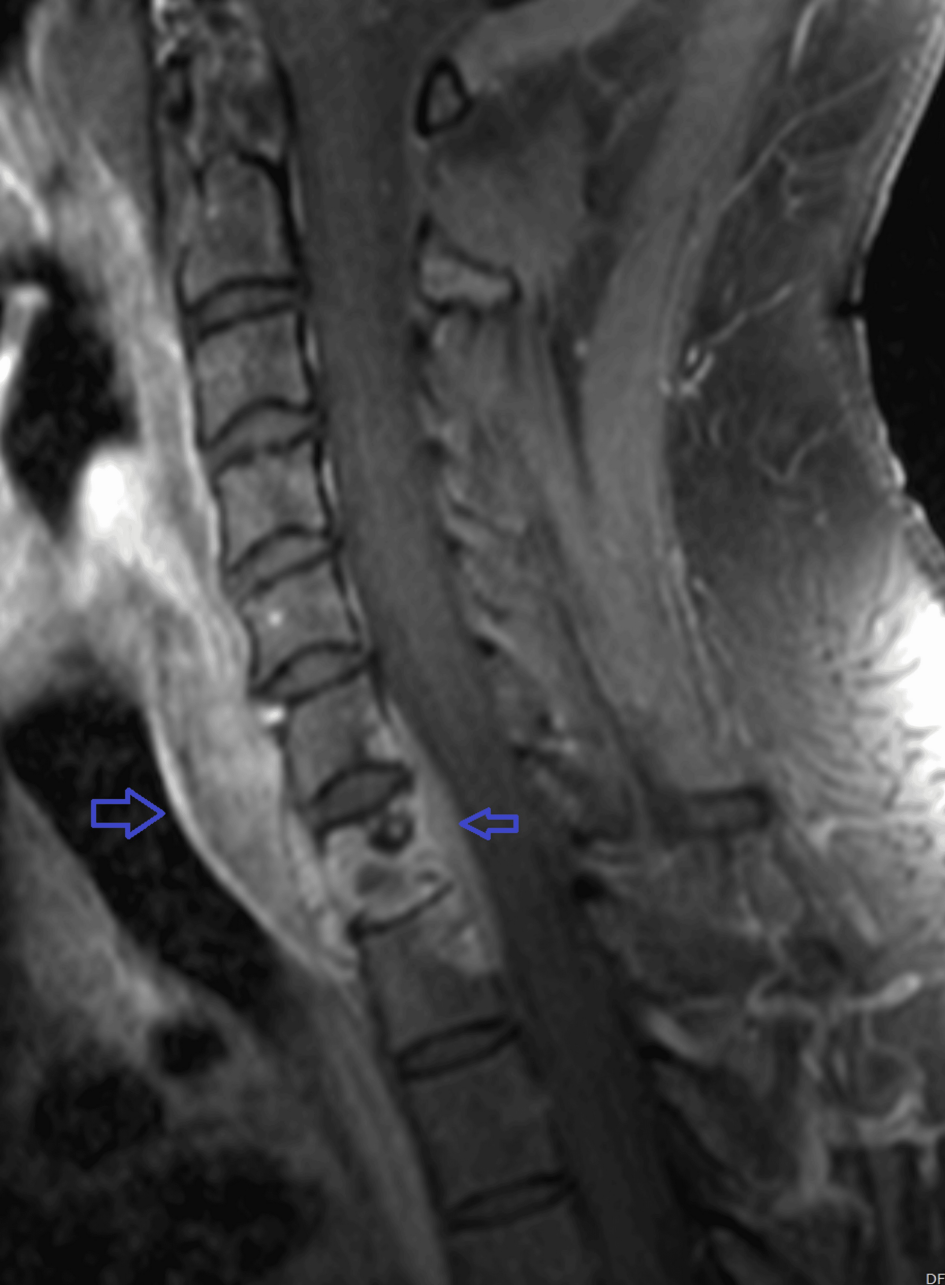
Sagittal T1 MRI showing C7 epidural abscess with involvement of the vertebral body and surrounding paravertebral soft tissues

A 33-year-old previously healthy Cameroon-origin man who lives in Tucson, Arizona, presented with persistent back pain and left hip pain. Initial outpatient imaging revealed lytic lesions in the sacrum, iliac bones, and the right eighth rib. A malignancy workup was initiated, and he underwent bone biopsy and bone marrow aspiration, which showed purulence with abundant Coccidioides spherules, but no malignancy or significant plasma cell proliferation. Serologic testing for coccidioidomycosis revealed complement fixation titers >1:256. He was admitted to the ICU for disseminated musculoskeletal coccidioidomycosis involving the left iliac bone, left sacrum, right iliac bone, and cervical spine (C6-T1), with evidence of discitis, osteomyelitis, and an anterior epidural phlegmonous collection (Figure 1).

**Figure 2:**
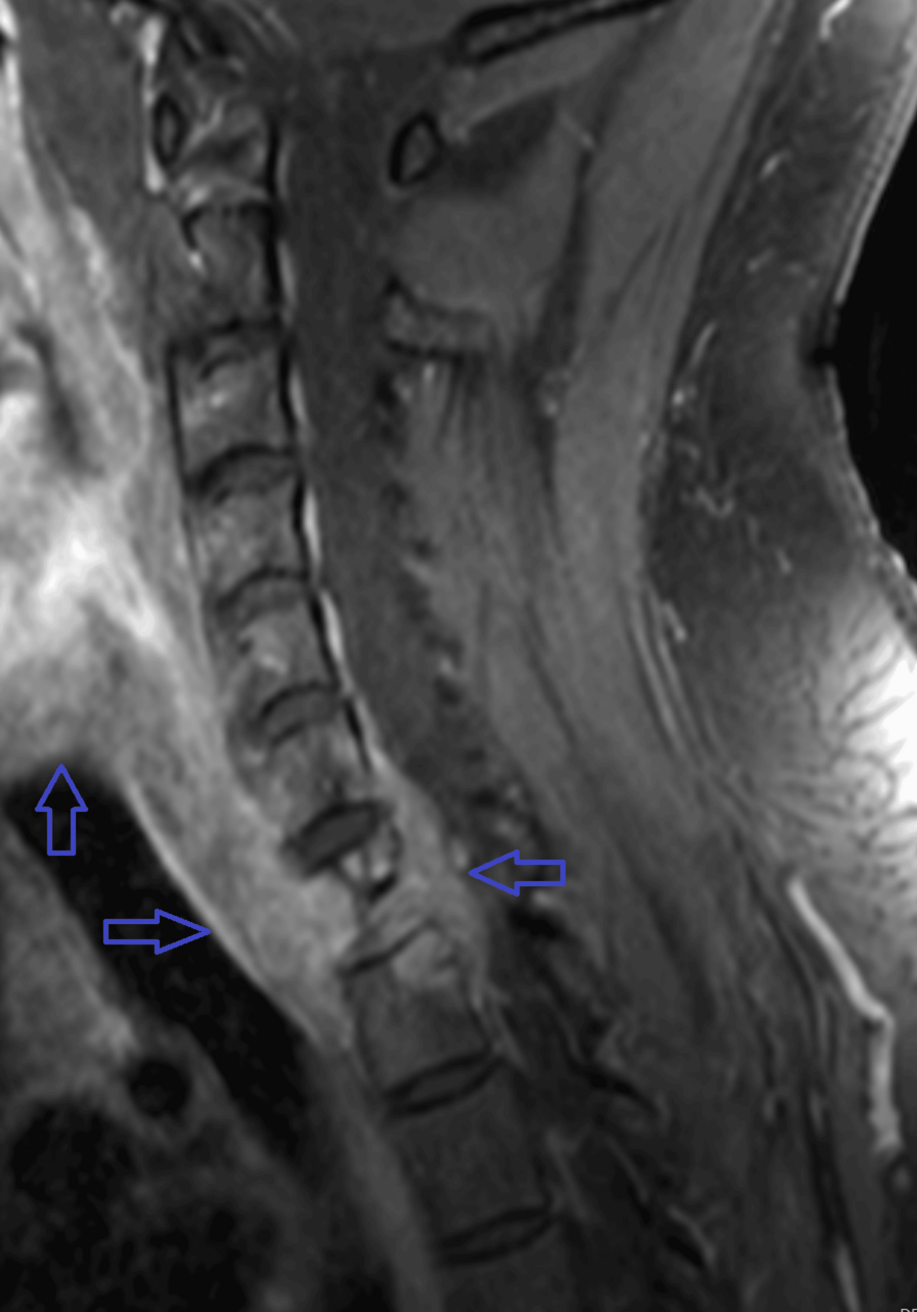
Repeat sagittal T1 post-contrast MRI demonstrating progression of the disease with near-complete destruction of the C7 vertebra due to disseminated coccidioidomycosis

**Figure 3:**
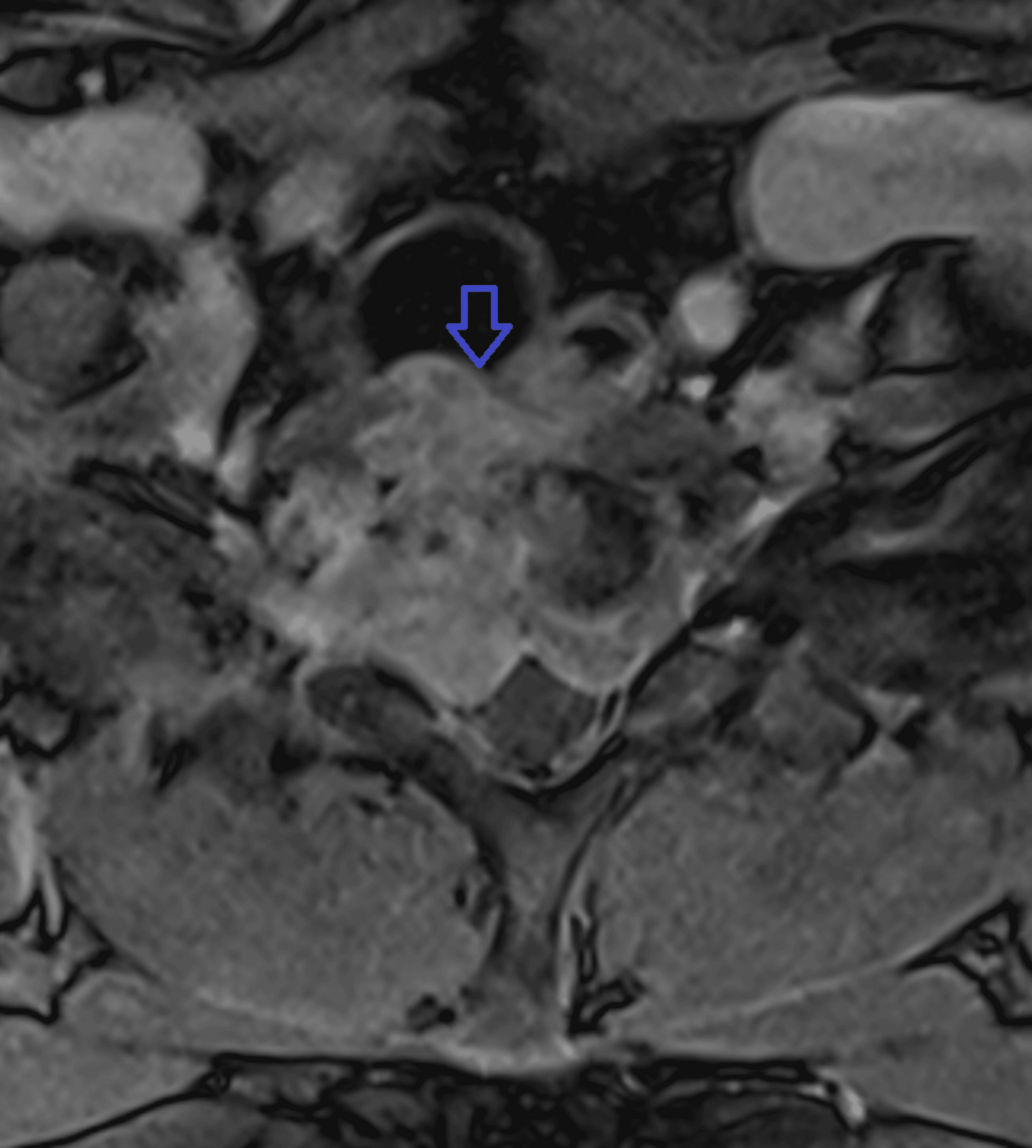
Repeat axial T1 post-contrast MRI demonstrating progression of the disease with near-complete destruction of the C7 vertebra due to disseminated coccidioidomycosis

**Figure 4:**
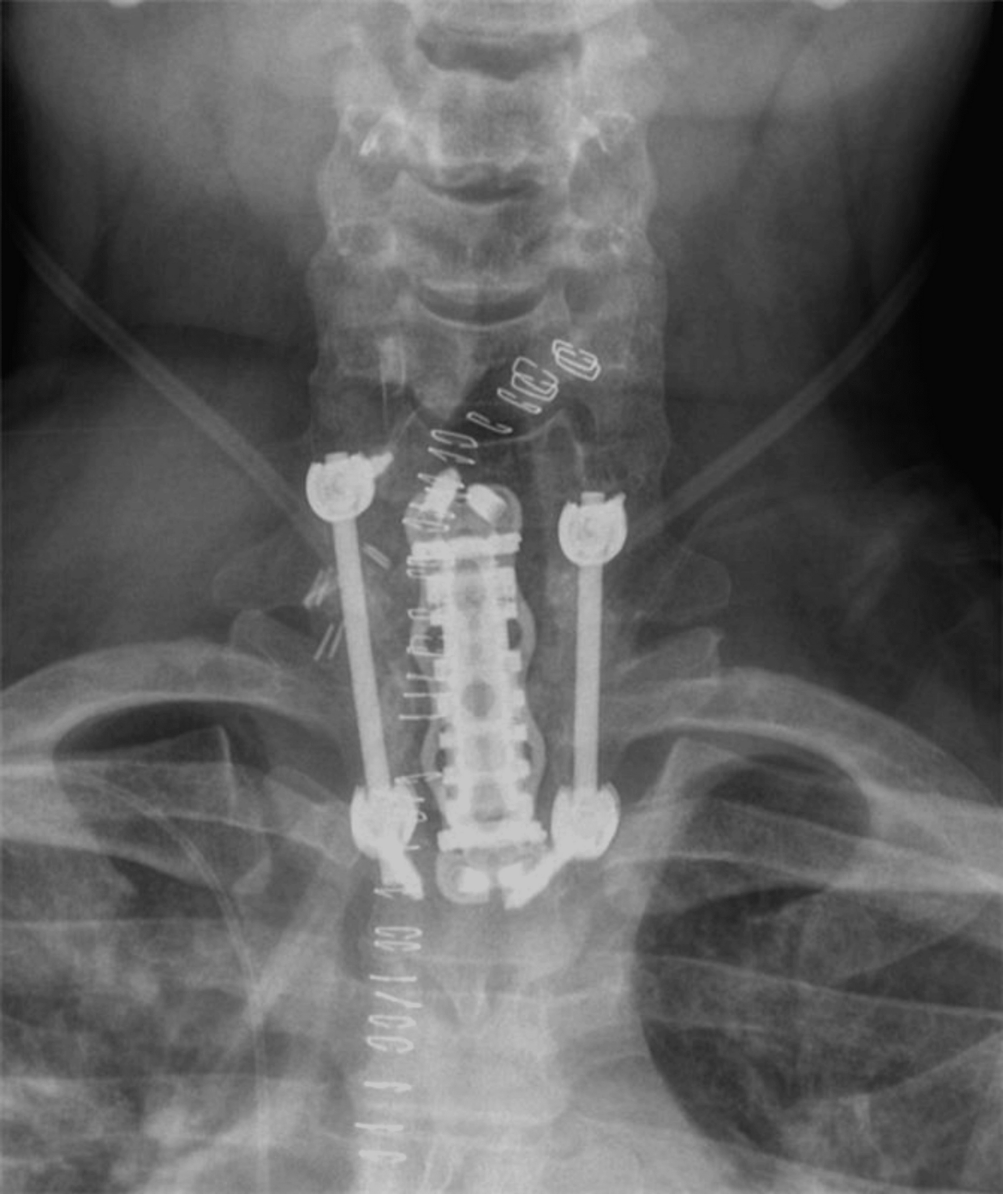
Post-operative CT spine demonstrating changes consistent with anterior cervical corpectomy and posterior cervical fusion

Initial treatment included IV amphotericin B and oral fluconazole. However, the patient developed acute kidney injury attributed to amphotericin nephrotoxicity. Renal function stabilized with dose reduction and IV fluids. Despite this, repeat MRI four weeks later revealed worsening osteomyelitis at C6-T1 with severe spinal canal stenosis (Figures 2, 3). Therefore, he underwent anterior cervical corpectomy at C7-T1 followed by posterior spinal fusion from C6 to T2 (Figure 4). He was extubated the following day and transferred to the medical floor.

The patient’s clinical course remained complex. On the sixth week, he underwent bilateral sacroiliac joint incision and drainage with antibiotic cement placement. Subsequently, he required lumbar spinal fusion and pelvic fixation. The antibiotic spacer was removed during a repeat debridement 10 days later. Due to elevated liver enzymes and hepatotoxicity, fluconazole was discontinued, and isavuconazole was initiated on the eighth week. Fosmanogepix therapy was later requested in anticipation of discharge.

After 10 weeks, the patient reported a slowly enlarging, painless right trunk lump, first noticed after his most recent surgery. CT imaging identified a 2 × 4 cm extra-thoracic chest wall mass with eighth rib involvement. Surgical excision was performed, with cultures revealing only scant mixed gram-positive flora, interpreted as contaminants; vancomycin was briefly initiated but later discontinued based on infectious disease consultation. Notably, his case remained complicated by persistent leukocytosis and thrombocytosis despite the absence of fever, with fluctuating transaminases (elevated ALT) due to mixed acute liver injury and alkaline phosphatase levels (463 IU/L; normal range: between 44 and 147 international units per liter (IU/L)) reflecting ongoing bone involvement. A soft tissue ultrasound and chest X-ray were ordered for further characterization of the chest wall swelling.

Throughout his hospitalization, the patient required substantial opioid analgesia, initially with IV Dilaudid PCA, later transitioned to scheduled and PRN oxycodone, along with muscle relaxants and neuropathic agents. Pain management follow-up was arranged in anticipation of tapering and long-term planning. Nutritional assessment confirmed protein-calorie malnutrition, and hepatology, orthopedics, infectious disease, and pain specialists were involved in his multidisciplinary care. Electrolyte derangements including hypokalemia, hypomagnesemia, and hyponatremia were managed per protocol. Anemia of chronic disease was present, and transfusion was reserved for hemoglobin <7 g/dL (normal range for men: 13.5-17.5 g/dL).

The patient remained hemodynamically stable, mobile without assistance, and neurologically intact. At the time of the most recent evaluation (14 weeks after hospitalization), IV liposomal amphotericin B (5 mg/kg daily) was discontinued and fosmanogepix therapy was approved. He was discharged on Tab. Isavuconazole (372 mg daily) and Tab. Fosmanogepix (800 mg daily) for an extended treatment course. He will follow up with the infectious disease clinic as an outpatient, with regular monitoring of liver function tests, renal function, and electrolytes.

## Discussion

Disseminated coccidioidomycosis with osseous involvement represents a severe and rare manifestation of *Coccidioides *infection, especially in immunocompetent patients [3,5]. The spine and pelvis are among the most commonly affected skeletal sites, and lesions can mimic metastatic malignancy or hematologic disorders, as was initially suspected in our patient. The diagnosis in this case was confirmed via biopsy, with histologic identification of Coccidioides spherules and supportive serologic and culture findings [10].

This case demonstrates the aggressive potential of disseminated coccidioidomycosis, as evidenced by the patient’s rapid progression to multilevel vertebral and pelvic osteomyelitis, severe spinal canal stenosis, and epidural phlegmon [3]. Surgical intervention is often necessary in such cases to prevent neurological compromise and to manage infectious collections [5]. Our patient underwent a series of orthopedic procedures including anterior and posterior cervical decompression and fusion, bilateral sacroiliac debridements, pelvic fixation, and removal of antibiotic spacers.

The antifungal management was also challenging. Amphotericin B, although effective, caused nephrotoxicity requiring careful monitoring and hydration. The hepatotoxicity associated with azoles led to the transition from fluconazole to isavuconazonium [11,12]. Fosmanogepix, a novel broad-spectrum antifungal agent, was pursued as salvage therapy given the extensive disease and limited options [13].

The patient’s African origin is noteworthy, as individuals of African or Filipino descent have been shown to be at higher risk for dissemination, even in the absence of traditional immunosuppressive risk factors [4,9]. The presence of persistent leukocytosis and thrombocytosis likely reflects a chronic inflammatory state, consistent with the ongoing fungal burden [14]. The elevated alkaline phosphatase and transaminases suggest active musculoskeletal infection and drug-induced liver injury, respectively [15].

Interestingly, the development of a chest wall mass with rib involvement in this patient may represent an unusual site of dissemination. Although cultures from the excised mass did not definitively confirm Coccidioides, the lesion’s clinical and radiographic features warranted surgical excision and close follow-up [3,5].

## Conclusions

Disseminated musculoskeletal coccidioidomycosis should be considered in patients with lytic bone lesions, especially in those from endemic areas or of high-risk ethnic backgrounds, regardless of immune status. Early biopsy, aggressive surgical debridement, and tailored antifungal therapy are essential for management. This case underscores the complexity of treating disseminated cocci involving the spine and pelvis, highlighting the need for a multidisciplinary approach. Long-term follow-up and antifungal suppression will likely be necessary. Newer agents like Fosmanogepix may offer hope for refractory cases.
